# Knowledge, attitudes, and practices related to spatial repellent for mosquito control and malaria risk perception in East Sumba, Indonesia

**DOI:** 10.1371/journal.pgph.0006929

**Published:** 2026-08-07

**Authors:** John T. Bandzuh, Kacey C. Ernst, Christopher K. Uejio, Jayleen K. L. Gunn, Salmon Pandarangga, Linda Rambu Kuba Yowi, Norlina Rambu Jola Kalunga, Sarah Hobgen, Kerry R. Cavanaugh, Mary H. Hayden

**Affiliations:** 1 Mount Aloysius College, School of Business, Arts, and Sciences, Department of Justice, Law, and Society, Cresson, Pennsylvania, United States of America; 2 University of Arizona, College of Public Health, Department of Epidemiology and Biostatistics, Tucson, Arizona, United States of America; 3 Florida State University, College of Social Science and Public Policy, Department of Geography, Tallahassee, Florida, United States of America; 4 Universitas Kristen Wira Wacana Sumba, East Sumba Island, Indonesia; 5 Kampung Raja, East Sumba Island, Indonesia; 6 London School of Hygiene and Tropical Medicine, London, United Kingdom; 7 University of Colorado, Lyda Hill Institute for Human Resilience, Colorado Springs, Colorado, United States of America; University of Oxford, UNITED KINGDOM OF GREAT BRITAIN AND NORTHERN IRELAND

## Abstract

While progress has been made in combating the global burden of malaria, Sumba Island, Indonesia, has trailed much of the country and region in reducing malaria infections. The study aimed to investigate local knowledge, attitudes, and practices related to mosquito control and malaria prevention in East Sumba Island, Indonesia. Primary data were collected from a cross-sectional household survey (n = 757) conducted across six subdistricts on East Sumba Island, Indonesia, stratified by urban and rural areas and high and low malaria incidence rates. Separate logistic and multinomial regression analyses examined survey responses on the acceptability of a novel spatial repellent, concern about malaria risk, and engagement in household and/or community mosquito control practices. The analysis considered differences by gender, education level, and socioeconomic position and controlled for respondents’ age and subdistrict. Respondents’ perceived risk of malaria transmission impacted their willingness to take action against mosquitoes by clearing tall grass and brush and educating others about mosquito control. Men were more willing to participate in all three actions related to clearing tall grass and brush (OR: 2.42, 95% CI: 1.63, 3.60, p-value: < 0.001), as well as educating others about mosquito control (OR: 1.73, 95% CI: 1.22, 2.45, p-value: 0.002). Older participants (50–59 years old) were more willing to sell and/or deliver mosquito control products in their community (OR: 2.05, 95% CI: 1.05, 4.00, p-value: 0.04). Study participants with average or above-average assets were more likely to be concerned about malaria transmission in their household over the next three months. Respondents were overwhelmingly interested in adopting a novel spatial repellent. Evidence from household surveys showed that respondents expressed interest in purchasing spatial repellents to combat malaria, demonstrating early buy-in for a novel technique to control mosquitoes. This research also revealed the role of gender in mosquito control and malaria prevention, specifically, willingness to clear tall grass or brush and to participate in mosquito-related education.

## Background

Despite advances in the fight against malaria, it remains a serious threat to the health of populations in endemic countries. In the World Health Organization’s South-East Asia region, seven of the nine malaria-endemic countries had reductions in malaria cases between 2015 and 2023 [1]. However, Indonesia, which accounts for nearly one-third of all malaria cases in the region, is one of only two countries that experienced an increase in malaria incidence during this same period [1]. As reported by Aisyah et al. (2024), the annual parasite index (API) increased from 0.99 per 1,000 population to 1.61 per 1,000 population between 2014 and 2022 [2]. This increase was at least in part due to the COVID-19 pandemic, which disrupted health services and related prevention strategies [2]. This reversal in progress suggests that Indonesia still has significant work to do to achieve its WHO-aligned Global Technical Strategy 2030 goal of eliminating malaria transmission by 2030.

Nationwide malaria control is complicated in Indonesia. With more than 6,000 inhabited islands, the distribution of resources and supplies is particularly challenging. Additional socioeconomic disparities and decentralized governance exacerbate these disparities in resource access [3]. National numbers belie the variable progress throughout the country as there continues to be significant heterogeneity in transmission within Indonesia. In recent years, some districts have eliminated or greatly reduced malaria transmission, while others remain highly endemic, particularly in eastern Indonesia [4,5]. Despite a reduction in the API of 13.7 per 1,000 population (2014) to 3.42 per 1,000 (2018), East Nusa Tenggara Province remains a challenging area in Indonesia to control malaria [6]. Furthermore, a recent study by Herdiana et al. (2025), found that the national incidence rate in Indonesia remained between 0.84 and 1.96 per 1,000 person-years from 2010 to 2025, yet the number of districts with no local malaria transmission has gradually increased [7]. Malaria and mosquito control remain major public health risks in Indonesia, particularly in the south and east.

Local variability in malaria endemicity, available resources, and cultural contexts necessitate collecting highly localized community knowledge of mosquito-borne diseases. Knowledge, attitudes, and practices (KAP) studies gauge public perception and knowledge and inform decision-makers on ways to improve control and prevention strategies [8–10]. Community perspectives are particularly important for implementing site-specific malaria prevention measures that align with local belief systems [6,11,12].

Sumba Island, in the East Nusa Tenggara Province of Indonesia, experiences high levels of malaria transmission. While numerous studies investigating mosquito ecology, malaria epidemiology, and studies on the efficacy of novel prevention strategies have been conducted on Sumba [6,13–16], little attention has been focused on community knowledge and mosquito control and/or disease prevention activities. A notable exception was a provincial survey that suggested malaria knowledge in the Sumba ethnic group was low compared to other ethnic groups within the East Nusa Tenggara Province [15]. Further investigation into the specific barriers to prevention and control is necessary to address the malaria burden in Sumba District [13,14,17]. Community knowledge provides insights into established malaria interventions, such as insecticide-treated nets (ITNs), as well as novel interventions, including spatial repellents (SR).

This study contributes to two gaps in the malaria literature: 1) it strengthens the evidence linking risk perception and household malaria protective actions, and 2) it provides market information on the acceptability of and willingness to pay for a novel SR. In the broader KAP literature, only a few studies have explicitly linked malaria risk perception to willingness to act on mosquito control efforts and/or malaria prevention [18]. A 2007 study in Malaysia found that study participants who perceived malaria as fatal were less likely to contract the disease [19]. Likewise, a 2013 study from Zanzibar found that the fear of increased cases in an area of low malaria transmission drove preventive behavior within the community [18]. The present study links perceived risk to a broader range of protective malaria actions while considering socioeconomic differences. Specifically, we examined the relationship between taking one or more actions to combat mosquitoes and the likelihood of stating that someone in the household was at risk of contracting malaria. This work will address the scarcity of research in the Sumba Island region by examining the connections between perceived risk and attitudes and behaviors related to mosquito control.

This study also investigates perceptions of and willingness to adopt a novel SR. SR, such as coils, sheets, or mats, volatilize chemicals to interfere with the mosquito’s attraction to human hosts or inhibit feeding behavior [13]. At the time of this study, the SR was a novel mosquito control technique for East Sumba Island, Indonesia. This study aims to provide further insight into study participants’ willingness to purchase and/or use the SR. When combined with other mosquito control measures such as ITNs and indoor residual spraying (IRS), SR has the potential to lower the risk of malaria transmission by creating “safe zones” [20].

SRs have been tested in Indonesia since the early 2000s, demonstrating good efficacy against *Culex* and *Anopheles* [21]. Controlled trials conducted on West Sumba Island, Indonesia, from 2015 to 2018 found that the protective efficacy of SRs against malaria infection was approximately 27% [13]. In the current study, we examined acceptability and willingness to pay for a SR on the same island, but in East Sumba. S.C. Johnson & Son, Inc. produces the novel SR, which releases transfluthrin into the air without the use of heat or electricity [13]. To date, few studies have examined the use and efficacy of SR [17,22,23]. This research provides insight into participants’ willingness and acceptance of a novel product, contributing valuable information about risk perception, willingness to use SR, and behaviors related to mosquito control in East Sumba Island, Indonesia. SRs are particularly useful in the context of traditional Sumbanese houses, which have high thatched roofs, few walls, and significant openness. While the SR demonstrated protective efficacy [13], its prophylactic benefit is localized and will likely require multiple emanators to achieve household-level coverage, particularly in the home types common in the study area. A recent study by Syahrani et al. confirmed that SR has not accelerated resistance in our study area (Sumba Island) and remains a valuable addition to mosquito control tactics [23].

## Methods

### Study settings

Surveys were conducted in households within East Sumba Regency, East Nusa Tenggara, Indonesia. The prevalence of malaria in East Nusa Tenggara Province in 2018 was 1.99%, nearly five and a half times higher than the national prevalence of 0.37% [24]. The East Sumba Regency is home to less than 0.1% of Indonesia’s total population (approximately 277,000) and spans an area of 7,000 square kilometers. Agriculture is the predominant occupational sector in East Sumba, primarily comprised of corn and other tropical export commodities, such as copra.

The household survey sampled urban and rural subdistricts with high and low malaria incidence. Subdistrict level malaria case data were obtained for 2014 from the Health Department of East Sumba District (Jl. R. Suprapto No.22, Prailiu, Kec. Kambera, Waingapu, East Sumba, East Nusa Tenggara, Indonesia). Incidence was calculated using 2010 population census data at the sub-district level from Badan Pusat Statistik (Table 1). The regency’s capital, Kota Waingapu, was the only urban subdistrict with a relatively low annual malaria incidence (ranging from 39-53 per 1,000 people). The rural subdistricts of Kambera and Kanatang, located near the capital, also had relatively low annual malaria incidence rates (ranging from 22 to 70 per 1,000 people). The rural subdistricts of Umalulu and Wula Waijelu exhibited the highest annual malaria incidence (ranging from 166 to 518 per 1,000 people) and were located farthest from the capital.

**Table 1:** Description of subdistricts and annual malaria incidence at the time of data collection.

Subdistrict	Urban (U) or Rural (R) & High (H) or Low (L) Incidence	Distance from Kota Waingapu	Cumulative Annual Malaria Incidence		
			2012 incidence	2013 incidence	2014 incidence
Kota Waingapu	Urban; low incidence	1 KM	49 per 1000	39 per 1000	53 per 1000
Kambera	Rural; low incidence	5 KM	70 per 1000	22 per 1000	34 per 1000
Kanatang	Rural; low incidence	6 KM	No Data	No Data	25 per 1000
Lewa	Rural; low incidence	60 KM	32 per 1000	22per 1000	34 per 1000
Umalulu	Rural; high incidence	62 KM	259 per 1000	305 per 1000	166 per 1000
Wula Waijelu	Rural; high incidence	123 KM	327 per 1000	429 per 1000	518 per 1000

### Health Belief Model (HBM)

This research applied the Health Belief Model (HBM) to evaluate and understand study participants’ knowledge, attitudes, and behaviors related to mosquito control and malaria prevention. The HBM remains one of the most widely used theories in health behavior research to date [25,26]. This study focuses on the first two of the six HBM constructs: 1) perceived susceptibility is the subjective risk of an individual contracting malaria, and 2) the perceived benefits are an individual’s feelings of the effectiveness of potential actions to combat mosquitoes/malaria (e.g., eliminating breeding sites, clearing tall grass and brush). Our previous work [10] focused on perceived barriers – individuals’ feelings about obstacles to action, such as perceived efficacy, environmental and/or household-level factors, and possible time commitments related to preventive actions in mosquito control.

The HBM has been criticized for failing to adequately consider environmental and socioeconomic factors that either encourage or inhibit health behaviors [27]. This research aims to expand upon the HBM by considering birth sex, socioeconomic status, environmental, and spatial factors related to individuals’ health-related behaviors using the social science theory of situated knowledge [28]. Briefly, this theory states that all forms of knowledge reflect the conditions in which they are produced, including power relations, sociocultural background, location, etc. While this theory acknowledges that all knowledge is partial and incomplete, it also recognizes that the partiality of that knowledge is an asset to understanding local, situated experiences.

Specifically, this paper examines household labor, situated knowledge of vector control, disease prevention behavior, and household decision-making [29–31] using a mixed-methods approach.

### Inclusivity in global research

Additional information regarding the ethical, cultural, and scientific considerations specific to inclusivity in global research is included in the S2 Checklist (Inclusivity in Global Research Checklist).

### Data source and measurements

Household surveys were comprised of 56 questions, primarily focusing on KAP related to community mosquito control and malaria prevention. Table 2 reports the study themes, related questions, response type, and corresponding statistical analysis. The surveys included standard demographic information, such as gender and education. The surveys were identical except for one question (#45) about SRs, which included three versions of a picture depicting two local individuals holding the SR product. The different survey types were randomly implemented across the study area; however, approximately equal numbers of each type were used. Survey type A (n = 252) included an image of one local male and one local female, survey type B (n = 254) included two local males, and survey type C (n = 251) included two local females. Different survey photos were used to test for potential effect modification (gender bias) in SR acceptance.

**Table 2:** Household survey research questions with types of survey responses and analysis.

Question/Theme	Survey Response Type and [# respondents]	Analysis
*Questions related to the decision-making of mosquito control products*		
Below is a picture of a new control measure that could be hung in your house called Spatial Repellent. Evidence suggests it is likely safe and effective at keeping mosquitoes out of one 3 square meter room for about two weeks. How likely would you be to try the control measure?	Very Likely or Likely [712]/Neither Likely or Unlikely or Unlikely or Very Unlikely [32]*	Logistic
I can decide to buy a mosquito control product that would cost [6,000] Rupiah without discussing it with my spouse/partner.	Yes [498]/no [250]*	Logistic
*Questions related to the concern of malaria transmission*		
How likely do you think it is that someone in your household will get malaria within the next three months?	Very unlikely [14]; unlikely [144]; neither likely nor unlikely [106]; likely [381], very likely [105].	Linear
Is malaria a concern for the whole community?	Binary Yes [615]/no [87]*	Logistic
*Questions related to action(s) to control mosquitoes*		
Would you be willing to do any of the following? • Clear the grass and brush around your own compound? • Help a neighbor clear the grass and brush around your neighbor’s compound? • Assist the community in clearing the grass and brush around everyone’s compound?	0 to 2 [243] versus 3 [506] actions	Logistic
Would you be willing to do any of the following? • Clean up trash in public spaces? • Help get rid of the places mosquitoes breed in shared areas in the community on roads by digging ditches and covering standing water with soil? • Empty containers with water?	0, 1 or 2, 3 actions	Collapsed Multinomial
Would you be willing to do any of the following? • Educate the community about mosquito diseases?	No [360]/Yes [389]	Logistic
Would you be willing to do any of the following? • Sell mosquito control products? • Deliver mosquito control products to households?	0 [464] versus selling and/or delivering [286]	Logistic

* Unsure or refused responses were dropped from the analysis.

S.C. Johnson (Raid) developed Mosquito Shield™, a transfluthrin (pyrethroid) based passive emanator that emits for up to 30 days and protects three square meters of indoor space [32]. While a single SR does not protect an entire home, it provides additional protection alongside other mosquito control techniques or addresses gaps in mosquito control measures. The number of SR emanators needed to protect an entire home may have important implications for affordability and sustained use, as recurring costs and the replacement of SRs may limit long-term feasibility. This S.C. Johnson social corporate responsibility program partnered with the Bill & Melinda Gates Foundation to test their product in West Sumba Island, Indonesia [12]. The Bill & Melinda Gates Foundation aims to eradicate malaria by reducing the disease’s burden and investing in measures to accelerate its eradication.

**Fig 1:**
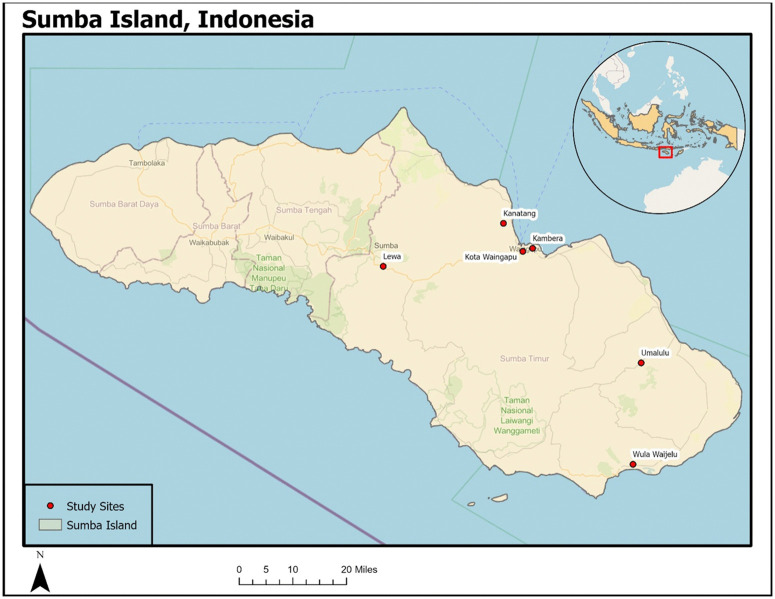
Map of study sites in East Nusa Tenggara Province, Sumba Island, Indonesia. *(Basemap data from* OpenStreetMap*)*.

A cross-sectional survey was constructed using *a priori* and emergent themes developed from KIIs and FGDs conducted in November 2015 [11]. Household surveys were field tested in both rural and urban sites and refined based on respondent feedback. The surveys were then conducted in 757 households from March 26 to April 5, 2016. The household survey was distributed across six subdistricts of East Sumba Regency, as shown in Fig 1. The number of surveys per district was proportional to the population’s size; 12% (n = 88) took place in Kanatang, followed by 13% (n = 98) in Wula Waijelu, 13% (n = 101) in Kambera, 17% (n = 130) in Kota Waingapu, 19% (n = 147) in Lewa, and 26% (n = 193) in Umalulu, and households were randomly selected to take part in this research. The surveys were conducted during the transition from the wet to dry season in the region; thus, the immediate risk of malaria declined during the time of data collection.

Trained, multilingual research assistants from the local university, Universitas Kristen Wira Wacana, field-tested the survey before initiation of the quantitative data collection. Revisions were made in collaboration with local researchers and the U.S. field team, and the questions were again field-tested to ensure their integrity. The field team administered the household surveys verbally in participants’ preferred language, either Sumbanese or Bahasa. Participants were included if they were at least 18 years of age, currently living with a partner, and had been residents of the subdistrict for at least six months before data collection. If domestic partners for a household were present during data collection, surveys were completed in a separate area of the property to limit the respondent’s influence on their partner’s responses. Because household-level decision-making related to mosquito control and disease prevention was at the center of this study, households with domestic partners were included in this research to assess decision-making dynamics. Written consent was obtained from participants prior to the household survey interview. The National Center for Atmospheric Research (NCAR) Institutional Review Board (#2015–16) approved the protocol. A memorandum of understanding was signed between NCAR and Universitas Kristen Wira Wacana, under which the latter deferred to NCAR’s approval of research protocols for this study.

The study separately examines participants’ concerns about malaria risk, engagement in household and/or community mosquito control practices, and acceptability and willingness to pay for an SR (Table 2). The mosquito control actions were classified into a) environmental risk factors such as clearing tall grass and brush (compound, neighborhood, or community), b) human-environment risk factors including larval mosquito source reduction and trash pick-up, c) mosquito-borne disease education, and d) behaviors related to mosquito control products, namely selling and/or delivering mosquito control products. These classifications provide a comprehensive sample of unique and widely accepted methods for combating mosquito-borne diseases.

Household survey questions that were examined and further analyzed in this research are located in Table 2. Respondents were asked to self-identify as either male or female. Educational attainment was categorized into five levels: no formal education, primary school, secondary school, tertiary education, and university or technical training.

The study utilized a reliable asset index, which estimates the value of possessions (e.g., cars, motorbikes, electricity, etc.), to measure household wealth. Asset-based indices are a useful measure of wealth in rural areas and have been used in previous malaria studies [33]. The values within the asset index were summed and classified into quantile categories: low wealth (0 – 624,500 Rupiah), lower-middle wealth (625,000 – 3,970,000 Rupiah), middle wealth (3,975,000 – 19,645,000 Rupiah), upper-middle wealth (19,650,000 – 23,140,000 Rupiah), and high wealth (23,145,000 – 251,475,000 Rupiah).

Urban/rural in this study refers to the distance from the capital and largest city on Sumba Island, Waingapu. The greater distance from the capital may indicate uneven geographic access to mosquito control and mosquito control institutions and initiatives [34]. Furthermore, having one or more children under the age of five controlled for access to healthcare and/or health-related information, as households are visited by *posyandu*. This community-based healthcare program provides information relevant to mosquito control and malaria [11,35,36]. The respondent’s age was grouped into sequential, non-overlapping 10-year age categories.

The analysis was conducted in R (version 3.5.2, 2018, Vienna, Austria) and RStudio (RStudio Team 2018, Boston, MA) [37,38]. All analyses considered differences by gender, education level, socioeconomic position, urban/rural, and having one or more children under the age of five. Furthermore, the analysis adjusted for the respondents’ age and subdistrict, and corresponding regression coefficients were omitted from the results table.

### Decision-making and mosquito control products

To gauge local perceptions of a novel SR product, study participants were asked whether they would use the specific SR and what they would be willing to pay for the product. Participants were separately asked if they would purchase mosquito control products for 6,000 Rupiah (USD 0.37) without consulting their partner. A 2006 study cited 6,000 Rupiah (USD 0.37) as the median cost of self-treatment for malaria [39]. This research question (#4) aims to identify household-level decision-making power and offer insight into the economic constraints households typically place on the price of mosquito control purchases. Study participants were presented with the same willingness to use SR questions, but with the aforementioned three gender variations of people holding the S.C. Johnson product. This logistic regression examined whether SR acceptance was modified by the respondent’s gender with a multiplicative interaction term.

### Risk perception and actions taken to combat malaria

Two survey questions were used to gauge study participants’ perceptions of malaria transmission risk within their households and communities. These questions were designed to examine perceived susceptibility to malaria within our HBM framework. Participants were asked about their concern about malaria for the whole community, as well as the “likelihood of someone in their household contracting malaria within the next three months.” Logistic and multinomial regression models were used to identify the characteristics of individuals who perceived malaria as a concern, along with associated sociodemographic and geographic factors.

### Actions to control mosquitoes

Questions about respondents’ willingness to take specific actions to control mosquitoes were used to examine perceived benefits of certain tactics in combating malaria within our HBM framework. Perceived benefits were assessed through survey questions regarding respondents’ willingness to engage in specific mosquito control practices, which served as indicators of their perceptions regarding the effectiveness of these preventive actions. Respondents were asked to check all that apply regarding their actions to control mosquitoes to combat malaria. Four mosquito control and malaria prevention subcategories were: 1) clearing grass and brush (mosquito habitat), 2) source reduction (oviposition sites), 3) education, and 4) selling and/or delivering mosquito control products. Willingness to take actions, such as clearing tall grass and brush or implementing source reduction, is linked to the perceived benefits of controlling mosquito populations and combating malaria, i.e., clearing grass and brush is designed to remove adult mosquito resting areas [40,41]. Multinomial regression was used to analyze actions to control mosquitoes (Table 2). Based on the response distributions, the analysis compared participants willing to participate in all three activities (n = 506) with those willing to participate in fewer than three activities (n = 243) involving grass and brush clearing. Source reduction (oviposition site elimination) often includes cleaning garbage within one’s property or neighborhood. While multiple studies examine educational campaigns, only Opiyo et al. (2007) investigated strategies to recruit others for these educational and/or prevention initiatives [42]. To date, no studies have examined study participants’ willingness to sell or deliver mosquito control products in their community in East Sumba to prevent malaria.

Several outcome variables derived from Likert-scale and “check-all-that-apply” questions were collapsed or regrouped for clarity and brevity. The study regrouped an action to the next closest category if it received fewer than 50 responses. Next, the grouping strategy considered logical splits due to the limited evidence published on this topic. For example, we considered grouping the actions into taking any action versus no action (removing breeding habitats; selling/delivering mosquito control products) or participating in all of the actions versus some/none of the actions (clearing grass and brush). This grouping strategy averaged over stratified effects of similar directions and magnitudes.

## Results

### Summary statistics

The household survey was conducted in East Sumba Island, Indonesia, with 757 participants. The demographic characteristics and descriptive statistics of the study sample are in S1 Table. Participants were primarily women (68.8%, n = 521). Most participants (53%, n = 401) were between 30 and 49 years old, and half (n = 377) of all respondents had attained at least a primary school education. Eighty-three percent (n = 627) of participants lived in rural areas compared to urban areas. Slightly more than half (52%, n = 392) of respondents had children under five years old living in their household and were eligible for *posyandu* healthcare visits, possibly providing respondents with information and/or products related to mosquito control and disease prevention.

### Decision-making and mosquito control products

The household survey revealed that 95% (n = 717) of participants were likely or very likely to try the S.C. Johnson & Son, Inc. SR described in the survey. There were no notable sociodemographic differences in respondents’ willingness to use the SR (S2 Table). For example, there were no significant differences for respondents over the age of 59 years old compared to those 20–29 years of age (OR: 2.04, 95% CI: 0.60, 6.92, p-value: 0.25). Next, we investigated the characteristics of participants who were more likely to purchase mosquito control products for 6,000 Rupiah (USD 0.37) without consulting their spouse or partner (S3 Table). Results indicate that 66.5% (n = 498) of respondents state they can purchase mosquito control products without consulting the other decision-maker within their household. Respondents with high educational levels (tertiary) had more autonomy in making this financial decision.

### Risk perception and actions taken to combat malaria

The survey gauged participants’ perceived risk and/or concern about malaria transmission. Participants were asked about the likelihood that someone in their household would contract malaria within the next three months (Table 3). Compared to the lowest wealth category, the middle (OR: 0.78, 95% CI: 0.62, 0.99, p-value: 0.05) and highest wealth (OR: 0.67, 95% CI: 0.50, 0.90, p-value: 0.01) study participants expressed less concern about contracting malaria in the subsequent months. Results were similar when participants were asked if malaria was a concern for the whole community (S4 Table).

**Table 3:** Concern of household-level malaria transmission from a simple linear regression model.

Variables	How likely do you think it is that someone in your household will get malaria within the next three months?		
	Odds Ratio	p-value	Standard Error
Age 30–39	0.93	0.63	0.15
Age 40–49	1.12	0.48	0.15
Age 50–59	0.92	0.60	0.16
Age 59+	1.14	0.44	0.17
Children under five years	1.07	0.33	0.08
Primary Education	1.00	0.99	0.13
Secondary Education	0.89	0.46	0.16
Tertiary Education	0.84	0.29	0.16
University Education	0.92	0.68	0.20
Lower-Middle Wealth	1.00	1.00	0.12
**Middle Wealth**	**0.78**	**0.05**	**0.12**
Upper-Middle Wealth	0.80	0.09	0.13
**High Wealth**	**0.67**	**0.01**	**0.15**
Urban	0.92	0.45	0.11
Gender (Male)	1.03	0.68	0.08

Statistical results were also adjusted for subdistricts.

### Actions to control mosquitoes

Results in Tables 4–6 summarize whether respondents’ concern for malaria altered their likelihood of participating in actions to control mosquitoes and/or combat malaria. For a) clearing grass and brush, b) source reduction, and c) selling and/or delivering mosquito control products, we initially ran multinomial statistical models. For clarity and brevity, we empirically regrouped the number of actions (based on the distribution of responses) and/or the similarity of statistical results, and reran the analysis. We first report the logistic regression results for participating in all three clearing grass and brush activities versus zero to two activities (Table 4). Participants who reported concern for household malaria transmission (OR: 2.71, 95% CI: 1.83, 4.01, p-value: < 0.001), men compared to women (OR: 2.42, 95% CI: 1.63, 3.60, p-value: < 0.001), and respondents with middle-level wealth (OR: 1.98, 95% CI: 1.14, 3.44, p-value: 0.02) were more likely to participate in multiple grass and brush clearing activities.

**Table 4:** Willingness to participate in all three actions (versus zero to two) related to clearing grass and brush habitats to control mosquitoes.

Variables	Willingness to participate in actions related to clearing grass and brush habitats to control mosquitoes		
	Odds Ratio	p-value	Confidence Interval
Age 30–39	1.45	0.25	0.77, 2.73
Age 40–49	1.40	0.31	0.73, 2.67
Age 50–59	1.15	0.68	0.59, 2.27
Age 59+	1.32	0.44	0.65, 2.71
Children under five years	1.19	0.32	0.85, 1.66
Primary Education	0.89	0.69	0.5, 1.60
Secondary Education	1.04	0.92	0.51, 2.13
Tertiary Education	0.72	0.37	0.35, 1.48
University Education	1.00	1.00	0.42, 2.42
Lower-Middle Wealth	1.55	0.10	0.92, 2.59
**Middle Wealth**	**1.98**	**0.02**	**1.14, 3.44**
Upper-Middle Wealth	1.10	0.74	0.63, 1.90
High Wealth	0.99	0.96	0.53, 1.84
Urban	0.72	0.15	0.46, 1.13
**Gender (Male)**	**2.42**	**< 0.001**	**1.63, 3.60**
Concerned about malaria*	**2.71**	**< 0.001**	**1.83, 4.01**
Not Concerned about Malaria	0.41	0.11	0.13, 1.23

Statistical results were also adjusted for subdistricts.

*Concern of malaria transmission within their household in the next three months (Yes).

**Table 5:** Willingness to participate in one to three action(s) versus no action(s) related to removing mosquito breeding habitats to control mosquitoes.

Variables	Willingness to participate in actions related to removing mosquito breeding habitats to control mosquitoes		
	Odds Ratio	p-value	Confidence Interval
Age 30–39	1.10	0.84	0.45, 2.69
Age 40–49	1.74	0.25	0.67, 4.54
Age 50–59	2.22	0.13	0.79, 6.24
Age 59+	1.43	0.50	0.50, 4.09
Children under five years	1.02	0.95	0.60, 1.73
Primary Education	1.21	0.63	0.56, 2.65
Secondary Education	2.31	0.14	0.76, 7.03
Tertiary Education	1.82	0.28	0.62, 5.39
University Education	2.53	0.29	0.46, 13.95
Lower-Middle Wealth	1.71	0.16	0.81, 3.62
Middle Wealth	1.29	0.51	0.61, 2.72
Upper-Middle Wealth	0.96	0.91	0.45, 2.04
**High Wealth**	**4.18**	**0.04**	**1.07, 16.27**
Urban	2.56	0.08	0.88, 7.43
Gender (Male)	1.07	0.83	0.59, 1.93
Concerned about malaria*	0.83	0.60	0.41, 1.66

Statistical results were also adjusted for subdistricts.

*Concern of malaria transmission within their household in the next three months (Yes).

**Table 6:** Willingness to participate in actions related to mosquito education to control mosquitoes.

Variables	Willingness to participate in actions related to mosquito education		
	Odds Ratio	p-value	Confidence Interval
Age 30–39	1.41	0.27	0.76, 2.59
Age 40–49	1.41	0.28	0.75, 2.62
Age 50–59	1.78	0.08	0.93, 3.44
Age 59+	1.26	0.51	0.63, 2.51
Children under five years	0.94	0.70	0.68, 1.29
**Primary Education**	**2.97**	**< 0.001**	**1.66, 5.30**
**Secondary Education**	**3.54**	**< 0.001**	**1.78, 7.03**
**Tertiary Education**	**4.05**	**< 0.001**	**2.01, 8.19**
**University Education**	**3.82**	**0.002**	**1.62, 9.00**
Lower-Middle Wealth	1.47	0.14	0.89, 2.42
**Middle Wealth**	**2.35**	**0.001**	**1.4, 3.96**
**Upper-Middle Wealth**	**1.78**	**0.04**	**1.04, 3.04**
**High Wealth**	**2.75**	**0.001**	**1.48, 5.10**
Urban	0.81	0.36	0.52, 1.27
**Gender (Male)**	**1.73**	**0.002**	**1.22, 2.45**
Concerned about malaria*	**1.97**	**< 0.001**	**1.32, 2.94**

Statistical results were also adjusted for subdistricts.

*Concern of malaria transmission within their household in the next three months (Yes).

Second, we discuss the results of actions to eliminate mosquito oviposition sites. Based on the similarities in sociodemographic differences identified in the multinomial analysis, we regrouped the final analysis into two categories: engaging in more than one action (1–3) [n = 679] versus none [n = 67]. We then reported the logistic regression results (Table 5). In contrast to clearing grass and brush, malaria concern was unrelated to the number of source reduction activities. Key findings suggest higher income respondents were more likely to engage in source reduction practices (OR: 4.18, 95% CI: 1.07, 16.27, p-value: 0.04), as found in Table 5.

Table 6 highlights the results related to a willingness to participate in actions aimed at mosquito control education. The respondents’ concern about malaria increased their willingness to participate in mosquito education (OR: 1.97, 95% CI: 1.32, 2.94, p-value: < 0.001). Compared to other sociodemographic factors, study participants in the highest wealth categories (3–5) and men (OR: 1.73, 95% CI: 1.22, 2.45, p-value: 0.002) were more likely to be willing to educate others on controlling mosquitoes.

The sociodemographic characteristics of respondents willing to sell or deliver mosquito products were generally similar. Thus, we report the collapsed results of respondents willing to sell and/or deliver mosquito products compared to those who were unwilling to participate in this activity. Age influenced study participants’ willingness to sell and/or deliver mosquito control products (S5 Table). Older participants (50–59 years old) were significantly more willing to sell and/or deliver mosquito control products (OR: 2.05, 95% CI: 1.05–4.00, p = 0.04). Self-reported concern for malaria was unrelated to a willingness to sell or deliver mosquito control products in their community.

## Discussion

This study aimed to strengthen the evidence linking risk perception to household malaria protective actions and to provide information on acceptability and willingness to pay for a novel SR. We utilized the HBM to provide a framework for examining study participants’ perceived susceptibility to malaria and the perceived benefits of actions to control mosquitoes and prevent malaria. Perceived susceptibility to malaria was analyzed and reported in Table 3 and was subsequently operationalized as “concerned about malaria” in all analyses related to perceived benefits. Actions to control mosquitoes and combat malaria were reported in Tables 4–6. While several other studies have used the HBM to examine KAP related to mosquito control and malaria prevention, we aim to contribute to the literature on this framework in a malaria endemic region in Indonesia [43–46].

Overwhelmingly, study participants would be willing to adopt the SR from S.C. Johnson into their household-level control measures. This finding is notable, as the study area has lower access to routine IRS and long-lasting ITNs than other regions of Indonesia [12]. Several recent studies have noted positive results regarding perceptions and efficacy of Mosquito Shield SR [32,47–49]. While the SR demonstrated potential to reduce mosquito exposure, its protective effect is localized and may require multiple units to achieve meaningful household-level coverage, particularly in the larger or semi-open homes common in the study area.

Relatedly, participants were asked about their ability to purchase mosquito control products without consulting their spouse or partner. Results indicate that 66.5% of respondents reported having the power to single-handedly decide to buy mosquito control products valued at 6,000 Rupiah (USD $0.37). Other studies have investigated the household-level costs associated with mosquito control in South and Southeast Asia. A 2001 study in Thailand found that using nets, coils, and sprays cost up to $2.08 (USD) per month [50], with approximately 70% of households using such measures. Similar results were found in a 2007 study from India, which reported that study participants used “modern chemical methods with 92% usage in urban households and 64% usage in rural households, spending $2.20 (USD) and $1.60 (USD) monthly (respectively) [51].

While the SR demonstrated potential to reduce mosquito exposure, its protective effect is localized and may require multiple units to achieve meaningful household-level coverage, particularly in larger or semi-open homes common in the study area. This has important implications for affordability and sustained adoption, as the recurring costs and logistical demands of replacing or maintaining units may limit long-term feasibility in lower-resource settings. Accordingly, spatial repellents should be considered a complementary intervention rather than a standalone solution, and future implementation studies should further evaluate cost-effectiveness, household coverage requirements, and sustainability under real-world conditions.

Knowledge of malaria and familiarity with its transmission cycle are associated with perceived risk, prior infection, and other factors [9,52,53]. A KAP survey in Chetla, India, in 2018 revealed that most (65.6%) study participants believed malaria was a problem for their community [54], which was fewer than the reported number in the present study (81.2%). Older adults (50–59 years old), wealthier participants (wealth categories 3–5), men, and individuals who expressed concern about malaria were more likely to report willingness to participate in mosquito control and malaria prevention efforts.

Additionally, a study from Sub-Saharan African countries with endemic malaria concluded that policies focused on child and maternal health should be integrated into malaria prevention efforts [55]. This theme was also probed in related work in Indonesia [11].

Results from this study indicate that participants were more likely to clear grass and brush when they perceived their household to be at risk of malaria transmission. Multiple studies have found that risk perception and fear influence the willingness to take action to prevent malaria and control mosquitoes [18,56]. In Guyana, Aerts et al. (2020) found that the more fearful study participants were, the more likely they were to adopt preventive measures against vector-borne diseases. A 2013 study in Zanzibar found that fear of increased cases in an area of low malaria transmission drove preventive behavior within the community [18]. Other studies have discussed eliminating mosquito oviposition sites (source reduction) as an effective measure to control mosquitoes, particularly in terms of risk perception [57]. This could include, but is not limited to, improving individuals’ and communities’ understanding of mosquito-borne disease risk and an increased willingness to adopt methods of prevention and action to combat the disease(s). As found in these studies, fear and perceived risk play a role in understanding preventive behaviors and disease prevention. Furthermore, public health officials should consider these factors when communicating with communities.

Findings from this study revealed gender-related differences in willingness to participate in mosquito control and disease prevention activities. Men were significantly more likely to be willing to clear tall grass and brush and educate others compared to women. These differences may reflect sociocultural and behavioral patterns that influence mosquito exposure and levels of community engagement. In Sumba Island, gendered divisions of labor and daily routines result in men spending more time outdoors and away from their homes [58]. These factors could have important implications when designing or targeting participants for future mosquito control and malaria prevention strategies.

Knowledge of mosquitoes, economic class, gender, and age influences the type of mosquito and malaria control actions people may engage in. The households most concerned about malaria clear vegetation, while higher-income households prefer to hold on to higher social status roles, such as educating others. Few studies have investigated prevention activities taken to combat malaria in relation to wealth or education status. In Ethiopia, one study found that participants who were educated beyond the primary level believed malaria was preventable with source reduction (compared to others), which also signifies the importance of community education [46].

### Limitations

This study is subject to several limitations. There is a temporal gap between data collection and publication, though evidence suggests that the broader malaria epidemiological context and prevention landscape in the study area have remained relatively consistent [59]. While malaria incidence and prevention measures in Indonesia have evolved since data collection, the broader epidemiological structure, such as the geographic concentration of transmission in eastern provinces and the persistence of similar risk environments, has remained largely the same. Therefore, while the absolute burden may have shifted, the underlying dynamics captured in this study remain relevant; however, they should be interpreted with consideration of potential changes over time. Despite progress in mosquito control and malaria prevention, limitations remain (e.g., geographic barriers and COVID-19-related interruptions). We have supplemented our data collection with contemporary studies to contextualize and examine other work in the region regarding mosquito control and malaria prevention. This study focuses specifically on mosquito control techniques rather than broader malaria prevention measures, such as vaccination, which is not yet widely accessible within the study area [58]. While this study assessed participants’ willingness to adopt a novel SR, it did not evaluate other factors that may influence adoption decisions, such as participants’ prior knowledge of SRs or their perceptions of the intervention’s effectiveness and safety. Although the survey question described the SR as safe and indicated its duration of effectiveness, participants’ understanding of or attitudes toward these attributes were not assessed. The proportion of participants who replied unsure or refused for each question was generally a small range (0.3 to 4.6%). Nonetheless, the study presumes that unsure/refused responses were missing at random and did not differentially influence the statistical results.

### Future directions

Future directions for this work should include further examination of the role of gender in mosquito control decisions and perceptions of their effectiveness. Additionally, future studies should engage households that have adopted SR for continued use in West Sumba Island, Indonesia. Perceptions of continued efficacy, cost-effectiveness, and risk of use should be examined to determine the viability of long-term SR use as a commonplace control technique in the mosquito control landscape of Sumba Island, Indonesia.

## Conclusion

Perceptions and willingness to use mosquito control strategies are crucial determinants of community acceptance of such activities. A modified HBM framework was employed to investigate 1) novel techniques such as SR and associated monetary costs, 2) concern of malaria at the household and community levels, and 3) willingness to participate in mosquito control actions in East Sumba Island, Indonesia.

Results from this research revealed that participants expressed a high level of interest in using the novel SR product introduced in this study (95%). However, this expressed interest should be interpreted with caution, as it may reflect the novelty of the intervention or study context rather than sustained adoption in real-world settings. Despite using other mosquito control practices such as ITNs and IRS, study participants indicated a general willingness to adopt additional mosquito prevention strategies within their households or communities.

Survey results also uncovered the role of gender in the willingness to take multiple steps to control mosquitoes, specifically clearing tall grass and brush (OR: 2.71, 95% CI: 1.83, 4.01, p-value: < 0.001) and willingness to participate in mosquito control education (OR: 1.73, 95% CI: 1.22, 2.25, p-value: 0.002). These findings are valuable because this research is part of a larger study examining gender barriers in vector control. Actions such as clearing grass and brush, or a willingness to educate others, can help create bottom-up, community-driven mosquito control campaigns throughout Indonesia and beyond. At the same time, these results underscore the importance of situating SRs within a broader, integrated vector management framework rather than positioning them as a singular or inherently superior solution.

## Supporting information

S1 Checklist Inclusivity in global research. (DOCX)

S2 Checklist STROBE Checklist [60]. (DOCX)

S1 Table Demographic Characteristics of Study Sample. (DOCX)

S2 Table Willingness to use spatial repellent (SR) to control mosquitoes. (DOCX)

S3 Table Cost of using spatial repellent related to household-level decision making. (DOCX)

S4 Table Concern of malaria for the whole community. (DOCX)

S5 Table Willingness to participate in any action related to selling and/or delivering mosquito control products to control mosquitoes versus no action(s). (DOCX)

S1–S16 Data Shapefile Components. (ZIP)
