# Supplementary material for: Knowledge, attitudes, and practices related to spatial repellent for mosquito control and malaria risk perception in East Sumba, Indonesia
Source: PLOS Glob Public Health. 2026 Aug 7;6(8):e0006929. doi: 10.1371/journal.pgph.0006929 (PMC13450719; doi:10.1371/journal.pgph.0006929)
Supplement: Supplementary file 1 [file pgph.0006929.s001.docx]

**Supplemental Checklist 1 Modified STROBE Checklist —*cross-sectional studies***

|  | Item No. | Recommendation | Page  No. |
| --- | --- | --- | --- |
| **Title and abstract** | 1 | (*a*) Indicate the study’s design with a commonly used term in the title or the abstract | 1 |
|  |  | (*b*) Provide in the abstract an informative and balanced summary of what was done and what was found | 2-3 |
| Introduction | | | |
| Background/rationale | 2 | Explain the scientific background and rationale for the investigation being reported | 4-7 |
| Objectives | 3 | State the specific objectives, including any prespecified hypotheses | 6 |
| Methods | | | |
| Study design | 4 | Present key elements of the study design early in the paper | 2, 6 |
| Setting | 5 | Describe the setting, locations, and relevant dates, including periods of recruitment, exposure, follow-up, and data collection | 7-11 |
| Participants | 6 | (*a*) Give the eligibility criteria, and the sources and methods of selection of participants | 11 |
|  |  | (*b*) Clearly define all outcomes, exposures, predictors, potential confounders, and effect modifiers. Give diagnostic criteria, if applicable | 14-15 |
| Variables | 7 | For each variable of interest, give sources of data and details of methods of assessment (measurement). Describe the comparability of assessment methods if there is more than one group | 14-15 |
| Bias | 8 | Describe any efforts to address potential sources of bias | 10, 12 |
| Study size | 9 | Explain how the study size was arrived at | 10 |
| Quantitative variables | 10 | Explain how quantitative variables were handled in the analyses. If applicable, describe which groupings were chosen and why | 14-15 |
| Statistical methods | 11 | (*a*) Describe all statistical methods, including those used to control for confounding | 14-15 |
|  |  | (*b*) Describe any methods used to examine subgroups and interactions | 17-22 |
|  |  | (*c*) Explain how missing data were addressed |  |
| Participants | 12 | (*a*) Report numbers of individuals at each stage of study—e.g., numbers potentially eligible, examined for eligibility, confirmed eligible, included in the study, completing follow-up, and analysed | 10-11 |
|  |  | (*b*) Give reasons for non-participation at each stage |  |
| Descriptive data | 13 | (*a*) Give characteristics of study participants (e.g., demographic, clinical, social) and information on exposures and potential confounders | 17 |
|  |  | (*b*) Report numbers of outcome events or summary measures | 2-18 |

Continued on next page

| Outcome data | 14 | (*a*) Give unadjusted estimates and, if applicable, confounder-adjusted estimates and their precision (e.g., 95% confidence interval). Make clear which confounders were adjusted for and why they were included | 18-22 |
| --- | --- | --- | --- |
|  |  | (*b*) Report category boundaries when continuous variables were categorized | 13 |
| Key results | 15 | Summarise key results with reference to study objectives | 17-22 |
| Limitations | 16 | Discuss limitations of the study, taking into account sources of potential bias or imprecision. Discuss both the direction and the magnitude of any potential bias | 25-26 |
| Interpretation | 17 | (*a*) Give a cautious overall interpretation of results, considering objectives, limitations, multiplicity of analyses, results from similar studies, and other relevant evidence | 22-25 |
| Generalisability | 18 | (*b*) Discuss the generalisability (external validity) of the study results | 22-25 |
| **Other information** | | | |
| Funding | 19 | Give the source of funding and the role of the funders for the present study and, if applicable, for the original study on which the present article is based | 27-28 |
| Author contributions | 20 | Brief description detailing the role of the authors’ intellectual and/or technical contributions to the study | 28 |

*Modified from the following:*

von Elm, E., Altman, D. G., Egger, M., Pocock, S. J., Gøtzsche, P. C., Vandenbroucke, J. P., & the STROBE Initiative. (2007). The Strengthening the Reporting of Observational Studies in Epidemiology (STROBE) Statement: Guidelines for reporting observational studies. PLoS Medicine, 4(10), e296. 10.1371/journal.pmed.0040296.
