# Supplementary material for: Knowledge, attitudes, and practices related to spatial repellent for mosquito control and malaria risk perception in East Sumba, Indonesia
Source: PLOS Glob Public Health. 2026 Aug 7;6(8):e0006929. doi: 10.1371/journal.pgph.0006929 (PMC13450719; doi:10.1371/journal.pgph.0006929)
Supplement: Supplementary file 3 [file pgph.0006929.s003.docx]

**Supplemental Table 1 Demographic Characteristics of Study Sample**

| Characteristics |  | Frequency (n = ) | Percentage (%) |
| --- | --- | --- | --- |
| Age | Age 20-29 | 61 | 8.08 |
|  | Age 30-39 | 191 | 25.30 |
|  | Age 40-49 | 210 | 27.81 |
|  | Age 50-59 | 164 | 21.72 |
|  | Age 59+ | 129 | 17.09 |
| Education | No Formal Education | 78 | 10.30 |
|  | Primary Education | 377 | 49.80 |
|  | Secondary Education | 104 | 13.74 |
|  | Tertiary Education | 134 | 17.70 |
|  | University | 64 | 8.45 |
| Wealth Category | Low Wealth | 120 | 15.85 |
|  | Lower-Middle Wealth | 183 | 24.17 |
|  | Middle Wealth | 155 | 20.48 |
|  | Upper-Middle Wealth | 150 | 19.82 |
|  | High Wealth | 149 | 19.68 |
| Dwelling Type | Urban | 130 | 17.17 |
|  | Rural | 627 | 82.83 |
| Gender | Male | 236 | 31.18 |
|  | Female | 521 | 68.82 |
| Household with children under five years | Yes | 392 | 51.99 |
|  | No | 362 | 48.01 |
