# Supplementary material for: Knowledge, attitudes, and practices related to spatial repellent for mosquito control and malaria risk perception in East Sumba, Indonesia
Source: PLOS Glob Public Health. 2026 Aug 7;6(8):e0006929. doi: 10.1371/journal.pgph.0006929 (PMC13450719; doi:10.1371/journal.pgph.0006929)
Supplement: Supplementary file 4 [file pgph.0006929.s004.docx]

**Supplemental Table 2 Willingness to use spatial repellent (SR) to control mosquitoes**

| Variables | How likely would you be to try the control measure [spatial repellent]? | | |
| --- | --- | --- | --- |
|  | Odds Ratio | p-value | Confidence Interval |
| Age 30-39 | 1.91 | 0.29 | 0.58; 6.33 |
| Age 40-49 | 1.96 | 0.29 | 0.56; 6.88 |
| Age 50+ | 2.04 | 0.25 | 0.60; 6.92 |
| Children under five years | 0.85 | 0.69 | 0.40; 1.84 |
| Primary Education | 0.97 | 0.96 | 0.26; 3.57 |
| Secondary Education | 0.45 | 0.28 | 0.11; 1.93 |
| Tertiary Education | 1.14 | 0.88 | 0.21; 6.26 |
| University Education | 1.68 | 0.68 | 0.14; 20.32 |
| Lower-Middle Wealth | 1.89 | 0.22 | 0.69; 5.20 |
| Middle Wealth | 2.42 | 0.13 | 0.77; 7.64 |
| Upper-Middle Wealth | 2.21 | 0.19 | 0.68; 7.21 |
| High Wealth | 1.42 | 0.60 | 0.38; 5.27 |
| Urban | 5.72 | 0.09 | 0.74; 44.22 |
| Gender (Male) | 1.22 | 0.65 | 0.52; 2.86 |
| *Statistical results were also adjusted for subdistricts.* | | | |
