# Supplementary material for: Knowledge, attitudes, and practices related to spatial repellent for mosquito control and malaria risk perception in East Sumba, Indonesia
Source: PLOS Glob Public Health. 2026 Aug 7;6(8):e0006929. doi: 10.1371/journal.pgph.0006929 (PMC13450719; doi:10.1371/journal.pgph.0006929)
Supplement: Supplementary file 5 [file pgph.0006929.s005.docx]

**Supplemental Table 3 Cost of using spatial repellent related to household-level decision making**

| Variables | I can decide to buy a mosquito control product that would cost 6,000 Rupiah (0.37 USD) without discussing it with my spouse/partner. | | |
| --- | --- | --- | --- |
|  | Odds Ratio | p-value | Confidence Interval |
| Age 30-39 | 0.66 | 0.22 | 0.34; 1.28 |
| Age 40-49 | 0.66 | 0.24 | 0.34; 1.31 |
| Age 50-59 | 0.71 | 0.33 | 0.35; 1.43 |
| Age 59+ | 0.64 | 0.23 | 0.31; 1.33 |
| Children under five years | 1.14 | 0.43 | 0.83; 1.56 |
| Primary Education | 1.10 | 0.72 | 0.65; 1.87 |
| Secondary Education | 1.35 | 0.36 | 0.70; 2.61 |
| **Tertiary Education** | **2.01** | **0.05** | **1.01; 3.99** |
| University Education | 1.50 | 0.35 | 0.65; 3.45 |
| Lower-Middle Wealth | 0.82 | 0.42 | 0.50; 1.34 |
| Middle Wealth | 0.75 | 0.28 | 0.45; 1.26 |
| Upper-Middle Wealth | 0.89 | 0.67 | 0.52; 1.53 |
| High Wealth | 0.75 | 0.37 | 0.41; 1.40 |
| Urban | 1.14 | 0.57 | 0.73; 1.80 |
| Gender (Male) | 1.27 | 1.34 | 0.90; 1.80 |
| *Statistical results were also adjusted for subdistricts.* | | | |
