# Supplementary material for: Knowledge, attitudes, and practices related to spatial repellent for mosquito control and malaria risk perception in East Sumba, Indonesia
Source: PLOS Glob Public Health. 2026 Aug 7;6(8):e0006929. doi: 10.1371/journal.pgph.0006929 (PMC13450719; doi:10.1371/journal.pgph.0006929)
Supplement: Supplementary file 6 [file pgph.0006929.s006.docx]

**Supplemental** **Table 4 Concern of malaria for the whole community**

| Variables | Is malaria a concern for the whole community? | | |
| --- | --- | --- | --- |
|  | Odds Ratio | p-value | Confidence Interval |
| Age 30-39 | 0.94 | 0.68 | 0.70, 1.26 |
| Age 40-49 | 1.12 | 0.45 | 0.83, 1.51 |
| Age 50-59 | 0.93 | 0.64 | 0.68, 1.27 |
| Age 59+ | 1.15 | 0.42 | 0.83, 1.59 |
| Children under five years | 1.09 | 0.27 | 0.94, 1.26 |
| Primary Education | 1.02 | 0.86 | 0.79, 1.32 |
| Secondary Education | 0.92 | 0.58 | 0.67, 1.25 |
| Tertiary Education | 0.87 | 0.41 | 0.64, 1.20 |
| University Education | 0.96 | 0.85 | 0.65, 1.43 |
| Lower-Middle Wealth | 0.99 | 0.92 | 0.78, 1.25 |
| **Middle Wealth** | **0.76** | **0.03** | **0.59, 0.97** |
| Upper-Middle Wealth | 0.81 | 0.11 | 0.63, 1.05 |
| **High Wealth** | **0.66** | **0.01** | **0.50, 0.88** |
| Urban | 0.76 | 0.07 | 0.57, 1.02 |
| Gender (Male) | 1.03 | 0.68 | 0.88, 1.22 |
| *Statistical results were also adjusted for subdistricts.* | | | |
