# Supplementary material for: Knowledge, attitudes, and practices related to spatial repellent for mosquito control and malaria risk perception in East Sumba, Indonesia
Source: PLOS Glob Public Health. 2026 Aug 7;6(8):e0006929. doi: 10.1371/journal.pgph.0006929 (PMC13450719; doi:10.1371/journal.pgph.0006929)
Supplement: Supplementary file 7 [file pgph.0006929.s007.docx]

**Supplemental Table 5 Willingness to participate in any action related to selling and/or delivering mosquito control products to control mosquitoes versus no action(s)**

| Variables | Willingness to participate in actions related to selling and/or delivering mosquito control products | | |
| --- | --- | --- | --- |
|  | Odds Ratio | p-value | Confidence Interval |
| Age 30-39 | 1.24 | 0.51 | 0.65, 2.34 |
| Age 40-49 | 1.43 | 0.28 | 0.75, 2.73 |
| **Age 50-59** | **2.05** | **0.04** | **1.05, 4.00** |
| Age 59+ | 1.56 | 0.21 | 0.77, 3.16 |
| Children under five years | 0.99 | 0.94 | 0.72, 1.35 |
| Primary Education | 1.14 | 0.63 | 0.67, 1.94 |
| Secondary Education | 0.94 | 0.86 | 0.49, 1.81 |
| Tertiary Education | 1.38 | 0.33 | 0.72, 2.66 |
| University Education | 1.31 | 0.51 | 0.58, 2.94 |
| Lower-Middle Wealth | 1.33 | 0.26 | 0.81, 2.21 |
| Middle Wealth | 1.56 | 0.09 | 0.93, 2.63 |
| Upper-Middle Wealth | 1.42 | 0.09 | 0.63, 1.03 |
| High Wealth | 1.55 | 0.21 | 0.83, 2.43 |
| Urban | 0.94 | 0.77 | 0.61, 1.44 |
| Gender (Male) | 0.96 | 0.79 | 0.68, 1.34 |
| Concerned about malaria* | 1.34 | 0.16 | 0.89, 1.99 |
| *Statistical results were also adjusted for subdistricts.*  **Concern of malaria transmission within their household in the next three months (Yes)* | | | |
