# Supplementary material for: Knowledge, attitudes, and practices related to spatial repellent for mosquito control and malaria risk perception in East Sumba, Indonesia
Source: PLOS Glob Public Health. 2026 Aug 7;6(8):e0006929. doi: 10.1371/journal.pgph.0006929 (PMC13450719; doi:10.1371/journal.pgph.0006929)
Supplement: Supplementary file 8 [file pgph.0006929.s008.zip › S16_Data.pdf]

INFORMED CONSENT / KESEPAKATAN SURVEY

Terima kasih telah meluangkan waktu berbicara dengan kami. Proyek ini dilakukan bersama tim dari National Center for Atmospheric Research, Universitas Arizona dan Universitas Wira Wacana. Kami berbicara dengan masyarakat di sini (Sumba Timur) mengenai berbagai cara untuk mengurangi nyamuk keluarga dan lingkungan anda. Kami akan menanyakan Anda (bapa/mama) beberapa pertanyaan mengenai cara apa yang digunakan untuk mengusir nyamuk dan serangga lainnya dan cara apa yang dianggap paling efektif. Kami juga akan menanyakan kamu beberapa pertanyaan terkait bagaimana Anda memutuskan cara apa yang harus dilakukan untuk mem membasmi nyamuk dan siapa yang membuat keputusan tersebut.

Saya akan menanyakan beberapa pertanyaan tentang hal-hal tersebut.

Kita akan berbicara sekitar 30 menit – atau lebih tergantung Anda dan waktu yang tersedia. Saya akan menyediakan waktu diakhir apabila Anda ingin menyakan sesuatu.

Percakapan ini bersifat rahasia – yang artinya bahwa apa yang kamu katakan kepada saya, tidak akan saya bicarakan kepada orang lain.

Kami akan menulis laporan tentang pembicaraan (diskusi) kita dan mungkin menyangkut beberapa pemikiran yang kamu berikan, tetapi kami tidak akan menuliskan namamu ataupun identitas kamu yang lainnya.

Kami akan mencatat dan merekam apa yang Anda bicarakan untuk memudahkan kami mengingat isi pembicaraan ini.

Keseluruhan pembicaraan ini bersifat sukarela. Karena itu, jika ada pertanyaan yang tidak ingin Anda jawab, maka silahkan tidak perlu menjawabnya. Hal penting yang perlu diketahui bahwa “Tidak ada jawaban yang benar ataupun salah”. Demikian juga, Anda dapat beritahu kami, jika kamu ingin mengakhiri pembicaraan kita kapan pun atau kita dapat bicarakan pada lain waktu dan kesempatan.

Apakah bapa/mama tetap SETUJU untuk melanjutkan pembicaraan kita?

DICE INSTRUCTIONS / PETUNJUK MENGGUNAKAN DADU

Ketika Anda tiba pada tempat atau lokasi yang telah ditentukan di sebuah desa, lemparlah dadu. Jika dadu berangka ganjil (1,3,5) pergi ke arah kiri. Jika dadu berangka genap, pergi kearah kanan. Untuk desa-desa berikut ini:Matawai, Wangga, Mauliru, Lumbu Kore, Kuta, Lewa Paku, lakukan survey di setiap rumah ke-3 (tiga). Untuk desa-desa berikut ini: Pambotanjara, Lairuru, Watu Puda, Mutunggeding, Laipandak, Hadakamali, Mondu, Matawai Pawali, Bidi Hunga, lakukan survey di rumah ke-2(dua) Jika grup tim Anda tidak dapat menyelesaikan jumlah rumah yang telah ditugaskan di tempat atau lokasi yang telah ditentukan karena pemilik menolak melakukan survey atau karena pemilik rumah tidak berada dirumah, maka pergilah ke desa pengganti.

Accelerating to Equal: How to Open Doors for Women in Global Health

A. Survey Type (Do not fill this in): Jenis survey (Jangan diisi): A: ○ B: ○ C: ●

B. Name of Interviewer
Nama pewawancara

C. Date of Interview / Tanggal wawancara

YEAR/Thn /	MONTH/Bln		/	DAY/hari	
2016	⓪	⓪		⓪	⓪
	①	①		①	①
		②		②	②
		③		③	③
		④			④
		⑤			⑤
		⑥			⑥
		⑦			⑦
		⑧			⑧
		⑨			⑨

D. Village Name / Nama desa

○ Matawai [1]	○ Pambotanjara [2]	○ Mauliru [3]
○ Wangga [4]	○ Lairuru [5]	○ Watu Puda [6]
○ Lumbu Kore [7]	○ Mutunggeding [8]	○ Laipandak [9]
○ Hadakamali [10]	○ Kuta [11]	○ Mondu [12]
○ Lewa Paku [13]	○ Matawai Pawali [14]	○ Bidi Hunga [15]

E.

Team # / Nomor Tim	RT # / Nomor RT		House # / Nomor KK	
①	⓪	⓪	⓪	⓪
②	①	①	①	①
③	②	②	②	②
④	③	③	③	③
⑤	④	④	④	④
⑥	⑤	⑤	⑤	⑤
⑦	⑥	⑥	⑥	⑥
⑧	⑦	⑦	⑦	⑦
⑨	⑧	⑧	⑧	⑧
⑩	⑨	⑨	⑨	⑨

F. Gender

○ Male [1]/ Laki-laki ○ Female [2]/Perempuan

1. In which community groups do you participate? Check all that apply.
Bapa/mama ikut/terlibat/bergabung di kelompok apa saja?

- ◯ Women's/men's groups [1]/PKK,
- ◯ Micro-credit orgs [2]/koperasi, kredit bantuan pemerintah (smart, anggur merah, pnpm)
- ◯ Agricultural production groups [3]/gapoktan
- ◯ Other social networks [4]/arisan
- ◯ Religious study or other church gp. activities [5]/majelis, guru sekolah minggu, komisi perempuan
- ◯ Income generating activities [6]/kelompok tenun,
- ◯ Table banking [7]/koperasi simpan-pinjam
- ◯ I don't participate in any groups (8) (**SKIP to question 3**) /Kalo tidak terlibat na langsung no 3
- ◯ Refuse [9]/menolak
- ◯ None [10]/ tidak terlibat
- ◯ Other (Fill in the box)[11]:

2. Why did you join a group in your community? Check all that apply.
Mengapa Bapa/Mama ikut dalam kelompok itu? **(SKIP to question 4 after answering)**

- ◯ Gets me out of the house [1]/bisa keluar rumah
- ◯ Learn new perspectives [2]/belajar hal2 baru
- ◯ Improves standing in the community [3]/tamba wawasan
- ◯ Makes me feel better [4]/lebe enak di luar
- ◯ Helps me make friends [5]/banyak teman
- ◯ Gives me place to talk about my family [6]/bisa ngerumpi
- ◯ Chance to win money in the lottery [7]/dapat uang/undian
- ◯ Work together to make money [8]/cari uang bersama
- ◯ Refuse [9]/menolak
- ◯ Other, (Fill in the box) [10]

3. What keeps you from participating in community groups and activities? Check all that apply.
Kenapa Bapa/Mama tidak ikut kelompok?

- ◯ Children at home [1]/tdk ada jaga anak
- ◯ No interest [2]/tdk tertarik
- ◯ No resources [3]/tdk ada modal
- ◯ Keeps me away from my job [4]/sibuk urus kerja
- ◯ Against my religious beliefs [5]/bertentangan dgn keyakinan
- ◯ Keeps me away from my other duties [6]/banyak kerja lain
- ◯ Not enough free time [7]/tdk ada waktu
- ◯ My husband does not want me to go [8]/paitua (maitua) tdk kasi ijin
- ◯ Other family members do not want me to go [9]/keluarga lain tidak kasi ijin
- ◯ Refuse [10]/menolak
- ◯ Other, (Fill in box) [11]/lain isi sendiri

4. Are there any activities you participate in for which you get paid in Rupiah or goods?
Apakah ada kegiatan2 yang Bapa/Mama ikut, dapat uang atau barang?

- ○ No [0]
- ○ Yes, money [1]
- ○ Yes, goods [2]
- ○ Yes, both [3]
- ○ Refuse [4]/menolak

5. Have you ever been a leader in your community?
Apakah bapa/mama pernah jadi pemimpin dalam masyarakat?

○ No [0] ○ Yes [1] ○ Unsure [2]/Tidak yakin (tidak tegas) ○ Refuse [3]/menolak

6. Has anyone encouraged you to be a leader?
Apakah ada orang lain yang pernah mendorong bapa/mama jadi pemimpin?

○ No [0] ○ Yes [1] ○ Unsure [2]/Tidak yakin (tidak tegas) ○ Refuse [3]/menolak

7. Is it acceptable for a man to have a female supervisor?
Bagus tidak perempuan jadi pemimpin?

○ No [0] ○ Yes [1] **(SKIP to Question 9)**
○ Unsure [2]/Tidak yakin (tidak tegas) ○ Refuse [3]/menolak

8. Why isn't it acceptable for women to supervise men?
Kenapa tidak bagus?

9. Is malaria a concern for the whole community?
Apakah malaria jadi masalah seluruh masyarakat?

○ No [0] ○ Yes [1] ○ Unsure [2]/Tidak yakin (tidak tegas) ○ Refuse [3]/menolak

10. How likely do you think it is that someone in your household will get malaria in the next three months?
Kira-kira kemungkinan anggota rumah tangga Bapa/mama kena malaria dalam 3 bulan ke depan?

- ◯ Very unlikely [1]/sangat tidak mungkin
- ◯ Unlikely [2]/tidak mungkin
- ◯ Neither likely nor unlikely [3]/ragu-ragu
- ◯ Likely [4]/mungkin
- ◯ Very likely [5]/sangat mungkin
- ◯ Refuse [6]/menolak

11. Has anyone ever come to your house to do fogging/spraying?
Ada yang pernah datang semprot nyamuk di sini?

◯ No [0] ◯ Yes [1] ◯ Unsure [2]/Tidak yakin (tidak tegas) ◯ Refuse [3]/menolak

12. Can a woman become a fogger/ sprayer?
Bisa tidak, perempuan jadi tukang semprot nyamuk?

◯ No [0] ◯ Yes [1] **(SKIP to Question 14)**
◯ Unsure [2]/Tidak yakin (tidak tegas) ◯ Refuse [3]/menolak

13. Why couldn't a woman become a fogger/ sprayer?
Kenapa perempuan tidak bisa jadi tukang semprot nyamuk?

- ◯ No Interest [1]/tidak tertarik
- ◯ Too dangerous [2]/tarlalu bahaya na…
- ◯ Equipment too heavy [3]/alat semprot terlalu berat
- ◯ They shouldn't work [4]/memang perempuan tidak perlu kerja begitu
- ◯ They need to take care of the kids [5]/Dorang punya tugas jaga anak
- ◯ Cultural reasons say no [6]/adat tidak boleh
- ◯ Unsure [7]/tidak yakin
- ◯ Refuse [8]/menolak
- ◯ Other (Fill in box) [9]/lainnya… isi sendiri

Would you be willing to do any of the following: (Apakah Bapa/mama bersedia melakukan hal2 berikut ini):

14. Clear the grass and brush around your own compound?
Bersihkan rumput di lingkungan rumah

○ No [0] ○ Yes [1] ○ Unsure [2]/Tidak yakin (tidak tegas) ○ Refuse [3]/menolak

15. Help a neighbor clear the grass and brush around your neighbor's compound?
Bantu tetangga bersihkan rumput di lingkungan sekitar rumah tetangga

○ No [0] ○ Yes [1] ○ Unsure [2]/Tidak yakin (tidak tegas) ○ Refuse [3]/menolak

16. Assist the community in clearing the grass and brush around everyone's compound?
Bersama masyarakat kerja bakti bersihkan lingkungan

○ No [0] ○ Yes [1] ○ Unsure [2]/Tidak yakin (tidak tegas) ○ Refuse [3]/menolak

17. Clean up trash in public spaces?
Bersihkan sampah di tempat umum

○ No [0] ○ Yes [1] ○ Unsure [2]/Tidak yakin (tidak tegas) ○ Refuse [3]/menolak

18. Help get rid of the places mosquitoes breed in shared areas in the community on roads by digging ditches and covering standing water with soil?
Basmi sarang nyamuk di tempat2 umum dengan bersihkan selokan/got dan menutup genangan air.

○ No [0] ○ Yes [1] ○ Unsure [2]/Tidak yakin (tidak tegas) ○ Refuse [3]/menolak

19. Empty containers with water?
Mengosongkan kaleng2 dll bekas yang ada air

○ No [0] ○ Yes [1] ○ Unsure [2]/Tidak yakin (tidak tegas) ○ Refuse [3]/menolak

20. Educate the community about mosquito diseases?
Memberikan pemahaman kepada masyarakat tentang penyakit yang disebabkan oleh nyamuk

○ No [0] ○ Yes [1] ○ Unsure [2]/Tidak yakin (tidak tegas) ○ Refuse [3]/menolak

21. Sell mosquito control products?
Jual obat nyamuk

○ No [0] ○ Yes [1] ○ Unsure [2]/Tidak yakin (tidak tegas) ○ Refuse [3]/menolak

22. Deliver mosquito control products to households?
Antar obat nyamuk ke rumah tangga? (pengecer)

○ No [0] ○ Yes [1] ○ Unsure [2]/Tidak yakin (tidak tegas) ○ Refuse [3]/menolak

23. Would you be willing to take a job to control mosquitoes?
Mau tidak bekerja untuk basmi nyamuk?

○ No [0] ○ Yes [1] ○ Unsure [2]/Tidak yakin (tidak tegas) ○ Refuse [3]/menolak

24. It would be ok to have a job where I/my wife would leave home before the sun rises.
Bapa/mam boleh tidak kalo pagi2 buta mama keluar rumah untuk bekerja?

○ No [0] ○ Yes [1] ○ Unsure [2]/Tidak yakin (tidak tegas) ○ Refuse [3]/menolak

25. It is ok to have a job that would take me/my wife away from home overnight for training.
Bapa/mama boleh tidak kalo mama nginap d tempat lain untuk ikut pelatihan?

○ No [0] ○ Yes [1] ○ Unsure [2]/Tidak yakin (tidak tegas) ○ Refuse [3]/menolak

26. I/My wife can take a job that requires me/her to travel to another village on a regular basis.
Bapa/mama tidak keberatan kalau mama pergi bekerja ke tempat lain dalam waktu tertentu?

○ No [0] ○ Yes [1] ○ Unsure [2]/Tidak yakin (tidak tegas) ○ Refuse [3]/menolak

27. I can decide to buy a mosquito control product that would cost 6000 Rupiah without discussing with my spouse/partner.
Kalau bapa/mama beli obat nyamuk harga 6000 rupiah masih kasi tau suami/istri?

○ No [0] ○ Yes [1] **(SKIP to question 30)**
○ Unsure [2]/Tidak yakin (tidak tegas) ○ Refuse [3]/menolak

28. I can decide to buy a mosquito control product that would cost 3000 Rupiah without discussing with my spouse/partner.
Kalau bapa/mama beli obat nyamuk harga 3000 rupiah masih kasi tau suami/istri?

○ No [0] ○ Yes [1] **(SKIP to question 30)**
○ Unsure [2]/Tidak yakin (tidak tegas) ○ Refuse [3]/menolak

29. I can decide to buy a mosquito control product that would cost 1500 Rupiah without discussing with my spouse/partner.
Kalau bapa/mama beli obat nyamuk harga 1500 rupiah masih kasi tau suami/istri?

- ◯ No [0]
- ◯ Yes [1]
- ◯ Unsure [2]/Tidak yakin (tidak tegas)
- ◯ Refuse [3]/menolak

30. It is important for a woman to earn money for her household.
Perlu bagi perempuan cari uang untuk rumah tangganya

- ◯ No [0]
- ◯ Yes [1]
- ◯ Unsure [2]/Tidak yakin (tidak tegas)
- ◯ Refuse [3]/menolak

31. It is acceptable for you/women in your family to work outside the home in a paid position?
Bisa diterima tidak kalo perempuan dalam keluarga bapa/mama kerja di luar rumah cari uang

- ◯ No [0] (**SKIP to question 33**)
- ◯ Yes [1]
- ◯ Unsure [2]
- ◯ Refuse [3]/menolak
- ◯ I already do work outside the home [4]/saya sudah kerja memang di luar rumah

32. During what times should a woman work outside the home for money? Check all that apply.
Mulai kapan dalam hidupnya perempuan bekerja di luar rumah cari uang

- ◯ Before she has children [1]/sebelum punya anak
- ◯ All through her life [2]/sepanjang hidup
- ◯ After the children are able to care from themselves [3]/setelah anak2 besar
- ◯ Before she is married [4]/sebelum menikah
- ◯ Unsure / I do not know [5]/tidak tau
- ◯ Refuse [6]/menolak

33. Women in my family are able to get a loan to start their own business.
Perempuan dalam keluarga bapa/mama bisa pinjam uang untuk mulai usaha

- ◯ No [0]
- ◯ Yes [1]
- ◯ Unsure [2]
- ◯ Refuse [3]/menolak
- ◯ I already have gotten a loan [4]/saya sudah pinjam sebelumnya

34. **Women:** I would be supported by my family to start my own business.
(ditanyakan pada perempuan) : saya akan didukung keluarga untuk memulai usaha saya

- ◯ No [0]
- ◯ Yes [1]
- ◯ Unsure [2]
- ◯ Refuse [3]/menolak
- ◯ I already have by own business[4]/saya sudah punya bisnis

35. **Men:** I would support my wife in starting her own business.
(ditanyakan pada laki2) : saya akan mendukung istri saya memulai usahanya

- ◯ No [0]
- ◯ Yes [1]
- ◯ Unsure [2]
- ◯ Refuse [3]/menolak
- ◯ She already has her own business [4]/istri saya sudah punya usaha sendiri

36. A woman has the skills or the natural ability to start and run a business on her own.
Perempuan punya ketrampilan atau bakat alamiah untuk mulai dan menjalankan bisnisnya

◯ No [0] ◯ Yes [1] ◯ Unsure [2]/Tidak yakin (tidak tegas) ◯ Refuse [3]/menolak

37. It is more important for boys to graduate secondary school than girls.
Lebih penting bagi anak laki2 untuk lulus sekolah lanjutan dibanding anak perempuan

◯ No [0] ◯ Yes [1] ◯ Unsure [2]/Tidak yakin (tidak tegas) ◯ Refuse [3]/menolak

38. Women should take part in politics.
Perempuan perlu terlibat dalam politik (mencalonkan diri sebagai apalahhhh gitu, politik praktis)

◯ No [0] ◯ Yes [1] ◯ Unsure [2]/Tidak yakin (tidak tegas) ◯ Refuse [3]/menolak

39. Sons are the only thing a women can rely on in her old age.
Hanya anak laki-laki yang paling diharapkan oleh perempuan di masa tuanya

◯ No [0] ◯ Yes [1] ◯ Unsure [2]/Tidak yakin (tidak tegas) ◯ Refuse [3]/menolak

40. It is acceptable for a woman to question her husband's opinions.
Apakah boleh jika seorang perempuan mempertanyakan pendapat suaminya

◯ No [0] ◯ Yes [1] ◯ Unsure [2]/Tidak yakin (tidak tegas) ◯ Refuse [3]/menolak

41. A woman must talk to her husband about expenditures.
Perempuan harus memberi tau suaminya tentang pengeluaran rumah tangga

○ No [0] ○ Yes [1] ○ Unsure [2]/Tidak yakin (tidak tegas) ○ Refuse [3]/menolak

42. A woman should have her own money to use as she decides.
Perempuan perlu punya uang sendiri untuk digunakan sesuai keinginannya.

○ No [0] ○ Yes [1] ○ Unsure [2]/Tidak yakin (tidak tegas) ○ Refuse [3]/menolak

43. A husband should let his wife work outside the home if she would like to.
Suami perlu mengijinkan istrinya bekerja di luar rumah sepanjang dia inginkan

○ No [0] ○ Yes [1] ○ Unsure [2]/Tidak yakin (tidak tegas) ○ Refuse [3]/menolak

44. If it is a question of children's health, it is best to do whatever the father wants.
Jika menyangkut kesehatan anak, pendapat bapa itulah yang paling betul

○ No [0] ○ Yes [1] ○ Unsure [2]/Tidak yakin (tidak tegas) ○ Refuse [3]/menolak

45. Below is a picture of a new control measure that could be hung in your house called Spatial Repellent. Evidence suggests it is likely safe and effective at keeping mosquitoes out of one 3 square meter room for about two weeks. How likely would you be to try the control measure?

Gambar di bawah ini adalah alat pengusir nyamuk yang dapat digantung di rumah yang disebut "Pengusir Nyamuk". Terdapat bukti bahwa alat ini aman dan bisa mengusi nyamuk pada ruangan berukuran 3 persegi selama kira-kira 2 minggu. Kira-kira bapa/mama mau coba alat tersebut?

- ◯ Very unlikely [1]/sangat tidak mau
- ◯ Unlikely [2]/tidak mau
- ◯ Neither likely nor unlikely [3]/mungkin
- ◯ Likely [4]/mau
- ◯ Very likely [5]/sangat mau
- ◯ Refuse [6]/menolak

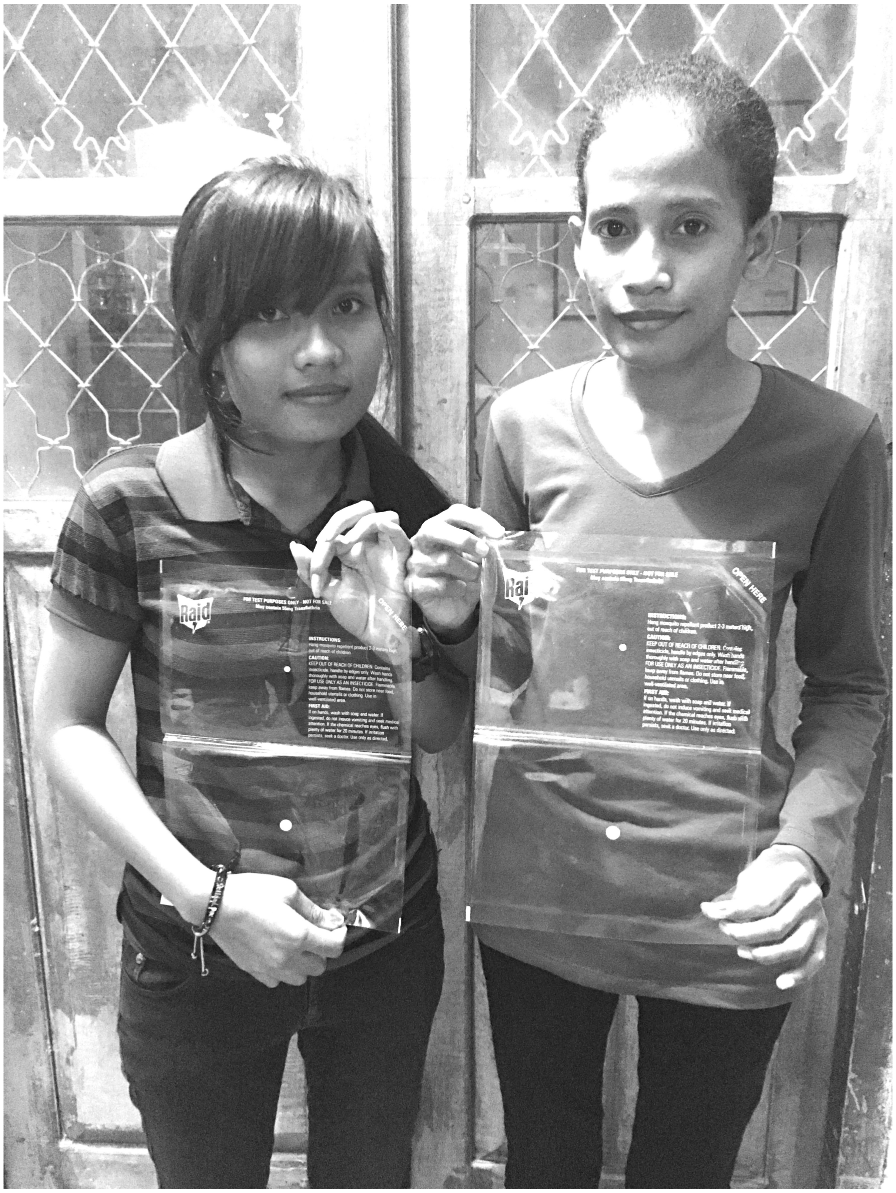

46. What is your age? Berapa umur bapa/mama?

⓪	⓪
①	①
②	②
③	③
④	④
⑤	⑤
⑥	⑥
⑦	⑦
⑧	⑧
⑨	⑨

47. What is your highest level of education? Pendidikan terakhir bapa/mama?

- ◯ Primary [1]/SD
- ◯ Secondary [2]/SMP
- ◯ Tertiary [3]/SMA
- ◯ Technical Training [4]/Akademi
- ◯ University [5]/Universitas
- ◯ Did not have chance to attend school [6]/Tidak pernah sekolah
- ◯ Refuse [7]/menolak

48. What ethnicity are you? Apa suku bapa/mama

- ◯ Sumbanese [1]/sumba
- ◯ Sabunese [2]/sabu
- ◯ Timorese [3]/timor
- ◯ Rotenese [4]/rote
- ◯ Balinese [5]/bali
- ◯ Javanese [6]/jawa
- ◯ Other (Fill in box) [7] [ ]
- ◯ Refuse [8]/menolak

49. Do you own any of the following? Apakah bapa/mama memiliki barang-barang seperti di bawah ini?

- ◯ Car [1]/mobil
- ◯ Motorbike [2]/sepeda motor
- ◯ Electricity [3]/barang elektronik
- ◯ Bicycle [4]/sepeda
- ◯ Television [5]/TV
- ◯ Cell phone [6]/hp
- ◯ Radio [7]/radio
- ◯ Pressure Lamp [8]/lampu gas
- ◯ Refuse [9]/menolak

50. What religion are you? Apa agama bapa/mama

- ◯ Christian [1]
- ◯ Catholic [2]
- ◯ Muslim [3]
- ◯ Traditional/marapu [4]
- ◯ None [5]/tidak ada
- ◯ Other (Fill in box) [6]
- ◯ Refuse [7]/menolak

51. How many children currently live with you?
Berapa orang anak (≤18 th) yang tinggal bersama bapa/mama sekarang ini?

0	1	2	3	4	5	6	7	8	9
10	11	12	13	14	15	16	17	18	19
20	21	22	23	24	25	26	27	28	29

52. How many of these children are under the age of 5? Berapa banyak balita di rumah?

0	1	2	3	4	5	6	7	8	9
10	11	12	13	14	15	16	17	18	19
20	21	22	23	24	25	26	27	28	29

THANK YOU / TERIMA KASIH

Do NOT ask the following: Jangan tanyakan hal2 di bawah ini:

53. Who else was present at the interview? Siapa lagi yang hadir pada saat wawancara

- ◯ Same age man [1]/beberapa laki2 sebaya
- ◯ Older man [2]/lelaki yang lebih tua
- ◯ Younger man [3]/(orang muda)
- ◯ Boy child [4]/anak laki2
- ◯ Girl child [5]/anak perempuan
- ◯ Older woman [6]/perempuan tua
- ◯ Younger woman [7]/perempuan muda
- ◯ Same age woman [8]/beberapa perempuan sebaya
- ◯ No one [9]/tidak ada orang selain yang diwawancarai

54. What was the attitude of the person who was being interviewed?
Waktu diwawancara, bagaimana sikapnya.

- ◯ Engaged and interested [1]/Terlibat dan tertarik
- ◯ Bored [2]/bosan
- ◯ Rude [3]/kasar
- ◯ Distracted [4]/merasa terganggu
- ◯ Drunk [5]/mabuk

55. Were there multiple wives in the house?
Apakah ada beberapa istri dalam rumah? (istri dari kepala keluarga yang disurvey)

◯ No [0] ◯ Yes [1] ◯ Unsure [2]/Tidak yakin (tidak tegas)

56. If so, which wife did you interview?
Jika demikian, istri keberapa yang diwawancarai?

- ◯ 1 [1]
- ◯ 2 [2]
- ◯ 3 [3]
- ◯ 4 [4]
- ◯ Unsure [5]/Tidak yakin (tidak tegas)
